# Long-term outcomes of SBRT for PSMA PET detected oligometastatic prostate cancer

**DOI:** 10.1186/s13014-023-02302-8

**Published:** 2023-08-01

**Authors:** Riche Mohan, A. Kneebone, T. Eade, E. Hsiao, L. Emmett, Christopher Brown, J. Hunter, G. Hruby

**Affiliations:** 1 Northern Sydney Cancer Centre, Royal North Shore Hospital, St Leonards, NSW 2065 Australia; 2 Northern Clinical School, University of Sydney, St Leonards, NSW 2065 Australia; 3 Garvan Institute of Medical Research, Darlinghurst, 2010 Australia

## Abstract

**Background:**

Oligometastatic disease in prostate cancer (PCa) is a challenging clinical scenario encountered more frequently with the widespread adoption of PSMA-PET. SBRT aims to defer androgen deprivation and may deliver sustained biochemical failure (BF) free survival in selected patients. Little long-term data is currently available regarding the effectiveness of this approach.

**Methods:**

A retrospective single institution study of PSMA-PET directed SBRT without initial ADT for oligo-metachronous PCa. Median dose/fractionation was 24 Gy in 2# to bones and 30 Gy in 3# to lymph nodes. The primary endpoint was time to BF (PSA + 0.2 ug/L above nadir). Secondary endpoints included time to ADT for relapse (i.e. palliative ADT), BF defined as PSA nadir + 2 ug/L, toxicity, patterns of failure and survival. Patients were excluded if they received ADT with their SBRT, had short disease-free interval, or > 3 metastases on PSMA-PET.

**Results:**

103 patients treated from November-2014 to December-2019 were analysed from our prospective database. Median follow-up was 5 years. 64 patients were treated for nodal only disease, 35 bone only and 4 mixed. 15% were free of any BF at 5 years with median time to BF of 1.1 years. 32% (33/103) of patients had further curative-intent radiation treatment following their first BF after SBRT, including subsequent SBRT. Eight patients underwent potentially curative treatment for their second or third relapse. Allowing for salvage treatment, 29/103 (28%) were biochemically disease free at last follow up. At 5 years, 39% of patients had never received any ADT and 55% had not started ADT for relapse with a median time to ADT for relapse of 5.5 years. There were 2 grade 3 toxicities (rib fracture and lymphoedema), and no local failures.

**Conclusion:**

PSMA-PET guided SBRT for oligo-metachronous PCa recurrence in appropriately triaged patients results in excellent local control, low toxicity and over 50% ADT free at 5 years.

## Introduction

Management of oligometastatic recurrence following definitive treatment for prostate cancer (PCa) is variable, with patient factors and clinician preference contributing to decision making. Clinicians are faced with a choice between observation, systemic therapy, metastasis-directed therapy (MDT), or a combination of these alternatives. This scenario has become more common with the widespread use of PSMA PET scans which enable earlier detection and delineation of oligometastatic PCa at much lower prostate-specific antigen (PSA) levels [1, 2].

Stereotactic Body Radiotherapy (SBRT) for MDT presents an attractive treatment option for this cohort of patients due to its non-invasive nature, excellent local control rates and limited side-effect profile [3–7]. Delaying androgen deprivation therapy (ADT) is an important outcome for these patients, given the treatment’s long-term side effects and poor patient tolerance[8, 9]. Furthermore, PSMA PET has the advantage of biologically directing therapy and excluding patients with more widespread disease at historically low PSA levels[10] as well as perhaps providing some prognostic information via PSMA PET intensity (SUVmax) measurement [11].

We previously reported the short-term outcomes of SBRT for PSMA detected oligometastatic prostate cancer[4] and demonstrated very high rates of local control, but the majority of our cohort had failed biochemically by 18months. This study aims to report the long-term experience of PSMA PET-guided SBRT for metachronous oligometastatic recurrence without ADT in a larger cohort and includes the use of sequential SBRT or curative intent conventionally fractionated re-treatment.

## Methods

This was a single centre observational study at an academic tertiary referral Hospital. Patients were extracted from an ethics approved prospective clinical research database.

Eligible patients had Gallium 68 PSMA PET detected oligometastatic prostate cancer (≤ 3 sites) treated with SBRT between November 2014 (date of routine access to PSMA scanning) and December 2019. Management of oligo-metastasis was not randomised, and patients treated with SBRT were likely to have a longer disease-free interval, PSA doubling longer than 6 months or had declined management with ADT. Patients were excluded if they were less than 6 months from their definitive treatment, had serum testosterone < 50ng/ml (i.e. castrate) or had received any neoadjuvant or concurrent androgen deprivation within 6 months of SBRT. No ADT was allowed at the time of initial SBRT, although short-term ADT (≤ 6 months) was allowed on conventionally fractionated (but not stereotactic) re-treatment.

The initial dose fractionation schedule for nodal disease was 50 Gy in 5 fractions (n = 16 patients) which was subsequently changed to 30 Gy in 3 fractions for the remaining 61 patients. Osseous disease received 24 Gy in 2 fractions for 90% of cases with 4 patients receiving 20 Gy in a single fraction. In the early period of the study, patients receiving the 5 fraction nodal approach had an expanded lower dose CTV as previously described by Kneebone et al[4]. In subsequent cases a PTV was created by generating a 5 mm expansion from the GTV as defined using PSMA PET and conventional imaging. Volumetric modulated arc therapy (VMAT) was used for all patients, treated at single centre with consistent voluming, planning setup and verification. Integrated cone beam CT was used to ensure correct setup and detect inter and intra -fraction movement.

### Treatment evaluation

Biochemical response was defined as any reduction in PSA following SBRT. Our Primary endpoint was Biochemical failure (BF) - defined as a 0.2 ug/l increase in PSA above the PSA nadir post SBRT. Even if patients were successfully salvaged with further SBRT or other curative treatment, they were still deemed to have had a biochemical failure if the above definition was met.

Secondary endpoints included clinical failure (defined by PSMA PET), initiation of (long term or intermittent) ADT for incurable relapse, secondary analysis using PSA Nadir + 2.0, subsequent treatment, local control and toxicity. If patients received further potentially “curative” radiation (pelvic nodal or prostate/prostate bed radiation) sometimes combined with short term (max 6 months) androgen deprivation (STAD), they were not regarded as having received ADT for relapse.

Subgroup analysis was performed to identify predictive features for biochemical failure (nadir + 0.2 definition) and ADT avoidance at 5 years post SBRT (both ADT for relapse and ADT-any).

### Statistical analysis

Statistical analysis was performed using R, version 4.0.2. Kaplan-Meir estimates were computed to estimate biochemical disease-free survival (bDFS) from the time of the first radiation treatment (RT). Cox regression models were used to identify predictive factors affecting biochemical failure and time to ADT relapse. Variables defined a priori were; median PSA at original diagnosis (< 9.6 vs. > 9.6 ng/ml); Gleason score (6 and 3 + 4 vs. 4 + 3 vs. 8+); time from diagnosis (< 5 vs. ≥ 5 year); site of metastasis (nodal (pelvic), nodal (other), mixed or bone alone); and number of metastases (1 vs. 2–3). As an exploratory analysis, PSA doubling time before SBRT was evaluated along with PSMA PET intensity or SUV Max (split by median). The 95% confidence interval (CI) was computed for all relevant estimates.

## Results

One-hundred and three (103) patients were treated with SBRT for metachronous oligometastatic disease between November 2014 and December 2019 (Table 1). The 103 patients had 120 sites treated with SBRT: 78 nodal and 42 bony sites.

**Table 1:** Demographics with effect on ADT free survival at 5 years – Univariate analysis

	N = 103^1^	%Risk ADT Free	N^1^	HR^2^	95% CI^2^	p-value	
**Age**			103			0.6	
< 60	28 (27%)	45% (29%, 69%)		—	—		
60–69	47 (46%)	56% (43%, 73%)		0.8	0.42, 1.53		
70+	28 (27%)	64% (47%, 87%)		0.68	0.32, 1.46		
**Treatment**			103			0.2	
RP + Adj RT	8 (7.8%)	— (—, —)		—	—		
Brachy	3 (2.9%)	— (—, —)		0.69	0.14, 3.44		
RP	35 (34%)	52% (38%, 72%)		0.48	0.19, 1.22		
RT alone	13 (13%)	38% (17%, 85%)		0.52	0.17, 1.56		
RP + Salvage RT	44 (43%)	70% (57%, 85%)		0.3	0.12, 0.76		
**Gleason**			97			0.4	
3 + 4	29 (30%)	69% (54%, 88%)		—	—		
4 + 3	30 (31%)	60% (45%, 80%)		1.41	0.66, 3.01		
8+	38 (39%)	43% (29%, 65%)		1.59	0.78, 3.28		
Unknown	6						
**Nodes**			103			0.6	
1	80 (78%)	58% (48%, 71%)		—	—		
2	19 (18%)	50% (31%, 80%)		1.06	0.51, 2.18		
3	4 (3.9%)	25% (4.6%, 100%)		1.87	0.58, 6.07		
**Doubling_time**			96			0.5	
< 6m	37 (39%)	50% (36%, 70%)		—	—		
6m+	59 (61%)	62% (50%, 77%)		0.82	0.46, 1.49		
Unknown	7						
**SiteOfMets**			103			0.9	
bone	35 (34%)	51% (36%, 72%)		—	—		
mixed	4 (3.9%)	50% (19%, 100%)		0.94	0.22, 4.06		
node (other)	8 (7.8%)	38% (15%, 92%)		1.05	0.39, 2.84		
node (pelvic)	56 (54%)	61% (49%, 76%)		0.79	0.44, 1.45		
**SUVmax_group**			93			0.4	
< 6.9	46 (49%)	58% (44%, 75%)		—	—		
6.9+	47 (51%)	57% (44%, 73%)		1.31	0.73, 2.36		
Unknown	10						
**YearsSinceDx**			103			0.3	
< 5	49 (48%)			—	—		
5+	54 (52%)			0.76	0.44, 1.31		
**PSA @ SBRT**	1.1 (0.5, 3.5)		103	1.03	0.97, 1.09	0.4	

^1^ n (%); Median (IQR)

^2^ HR = Hazard Ratio, CI = Confidence Interval

The median follow-up was 5 years. Median age at diagnosis was 65 years (43 to 82), and all but two patients had Gleason Score (GS) 7 or greater disease (Table 1). Most patients (87) had had a radical prostatectomy as their initial treatment. Twenty-four (24) of the 87 patients had received short term adjuvant or concurrent hormone therapy with their post-prostatectomy RT; and 3 of 13 with their definitive RT. Respectively, 14 (of 24) and 3 of 13 received concurrent prophylactic nodal irradiation. Median time from initial treatment to SBRT was 6 years.

**Fig. 1:**
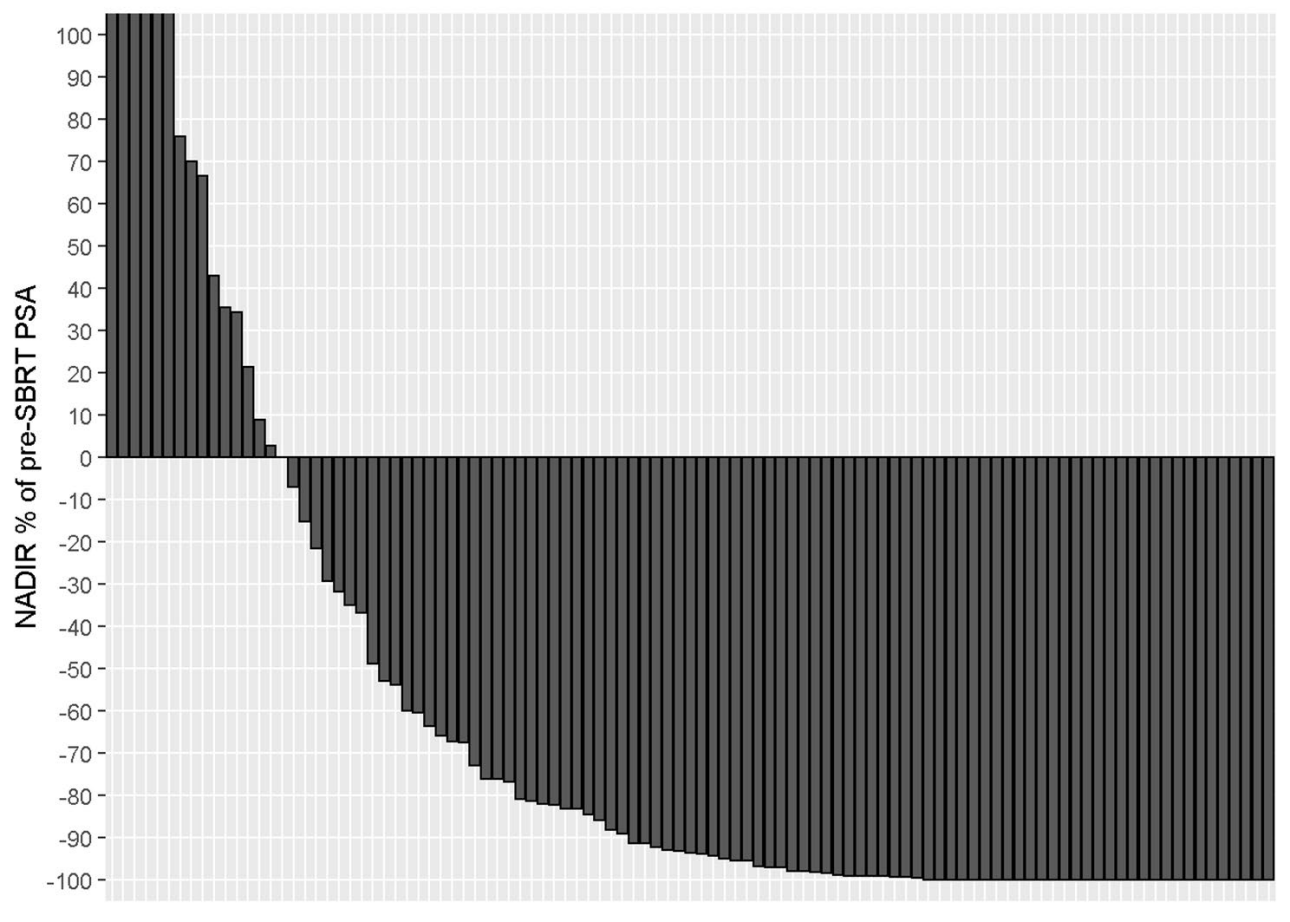
PSA Response to SBRT

Twenty-eight patients (27%) had a complete PSA response to SBRT. This has been depicted in the Waterfall plot, comparing pre and post SBRT PSA levels for the cohort (see Fig. 1).

**Fig. 2:**
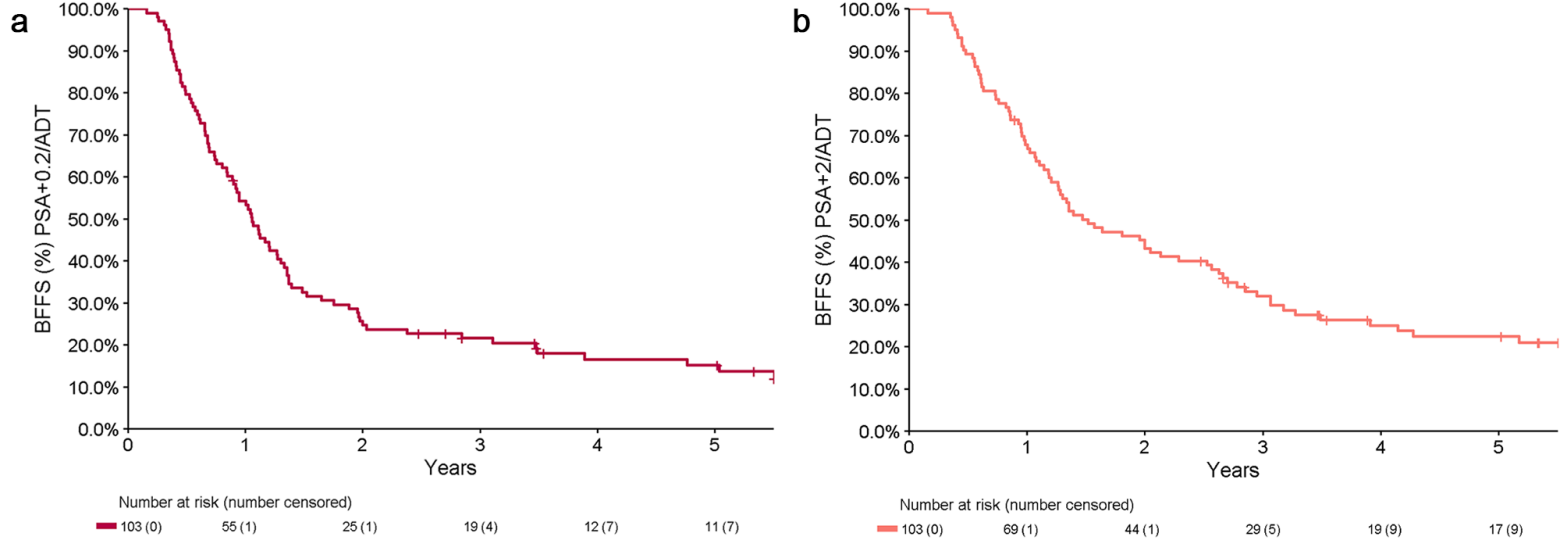
Time to Biochemical failure (**A**) defined as nadir + 0.2 (**B**) defined as nadir + 2

**Fig. 3:**
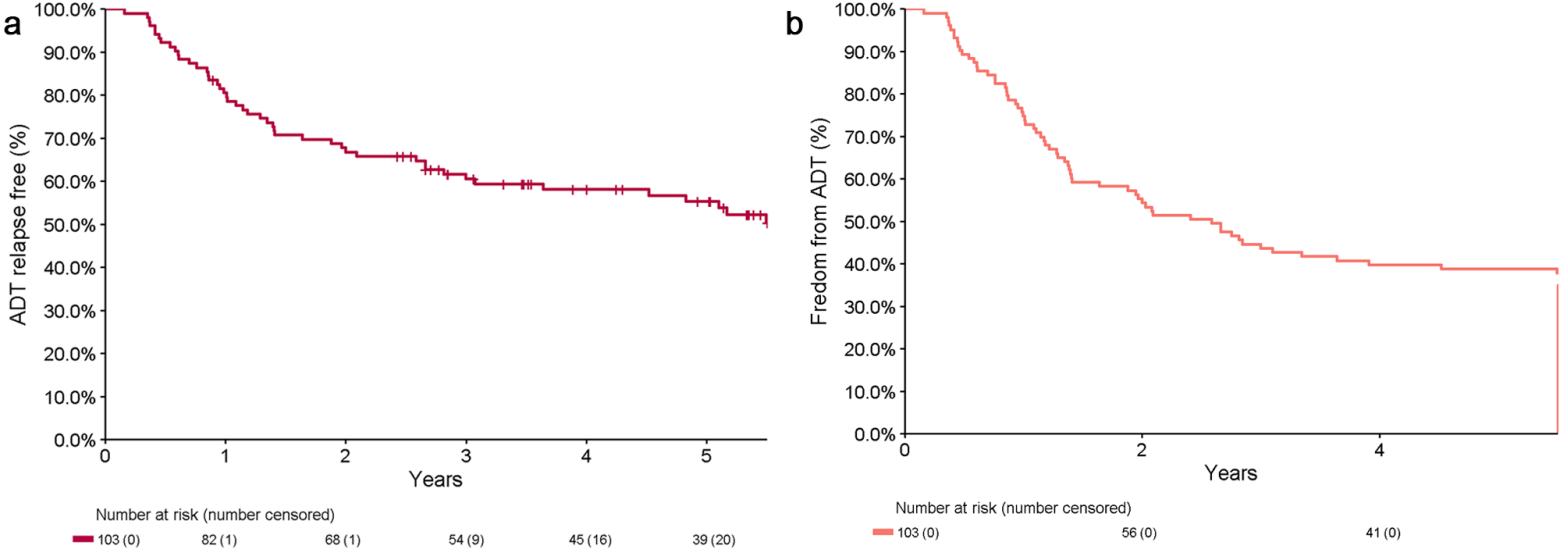
Time to ADT failure (**A**) ADT for Relapse (**B**) any ADT

Median time to biochemical failure (nadir + 0.2) was 1.1 years (0.90–1.3), with a 2-year BF free survival of 25% (18-35%) and a 5-year BF free survival of 15% (9.2-25%) (Fig. 2A). The a priori analysis of a nadir + 2 definition for biochemical failure demonstrated a median time to biochemical failure or commencement of ADT for relapse of 1.5 years (1.3,2.6) (Fig. 2B). Including subsequent MDT after initial SBRT, the 5-year ADT-free survival for the cohort was 55% (46-66%) for ADT relapse and 39% (30-49%) for any ADT (Fig. 3).

**Fig. 4:**
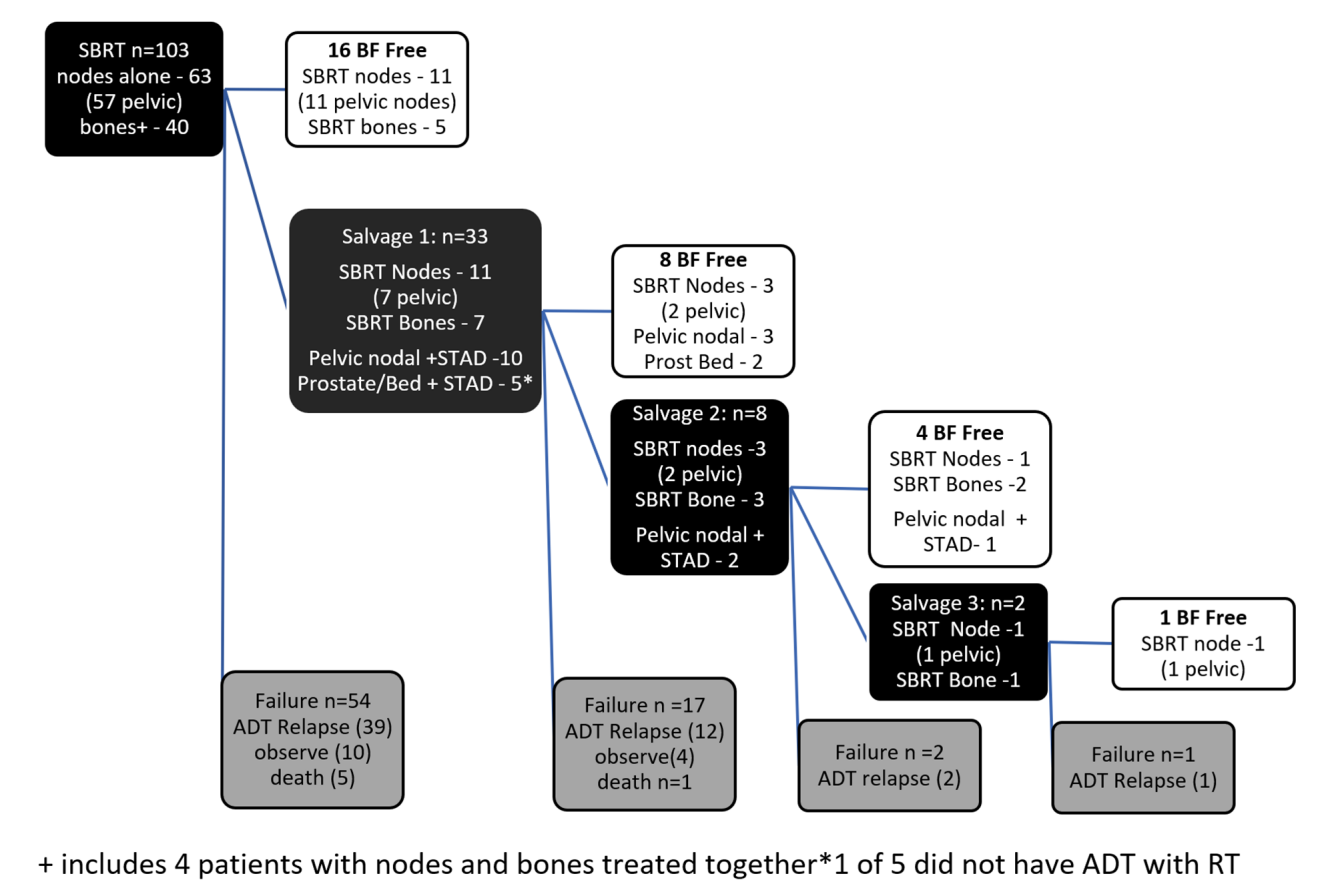
Cohort outcomes

Figure 4 outlines the patient journey at the time of analysis. The majority of patients who remain BF after initial SBRT had treatment to pelvic nodes (69%). Of the 63 patients who had nodal SBRT, 10 had received prior prophylactic pelvic nodal radiation as part of their prior management. Of the 87 patients who had a biochemical failure post SBRT, 33 had oligometastatic disease amenable to further “curative” radiation oncology intervention with 15 of these receiving concurrent short term androgen deprivation. In those undergoing further curative treatment, 13 (39%) were BF free (nadir + 0.2 definition) at time of analysis, however, longer follow up is required to assess sustained failure free survival in some cases. One patient underwent 4 sequential SBRT treatments (node-node-node-bone) resulting in ADT deferral for at least 6.3 years.

The median PSA at the time of commencement of ADT for relapse was 5 (Q1 = 2, Q3 = 9) with 77% commenced on ADT when PSA < 10 (Table 2). The median PSA of patients who have failed but remain under observation (14 in total) is currently 1.4 (range 0.14, 12) (Fig. 4).

**Table 2:** PSA at time of commencement for ADT for Relapse

Characteristic	N = 51^*1*^
PSA*	5 (2, 9)
Groups	
<2	12 (24%)
2–5	15 (29%)
5–10	12 (24%)
10+	12 (24%)

^*1*^ Median (Q1-Q3); n (%)

There were no significant risk factors for Biochemical Failure (nadir + 0.2 ng/L definition) or ADT relapse at 5 years (Table 1) on univariate analysis.

Post SBRT PSMA PET scans were available for 95 of the patients (92% of the cohort). Of these, 76 (80%) had a complete response in the treated lesions and the remaining showed partial or near complete response. There was no radiological evidence of any in-SBRT-field failure in this cohort.

There were no grade 4 toxicities reported. The most common low-grade patient reported toxicities were, short-term fatigue, or transient bowel or bladder irritation. Two patients experienced toxicity of note: 1 patient experienced persistent leg oedema (CTCAE Grade 2) following SBRT to a pelvic node with 50 Gy in 5 fractions, and 1 patient sustained a pathological rib fracture at the site of SBRT (24 Gy in 2 fractions). Six deaths were observed during the follow up period; 3 prostate cancer-related, 2 unknown and 1 unrelated to prostate cancer.

## Discussion

SBRT without ADT for PSMA PET detected oligometastatic prostate cancer in our cohort conferred a 15% 5-year biochemical failure free survival (nadir + 0.2ug/L definition), with 55% of patients not requiring palliative ADT before 5 years. The use of potentially curative subsequent salvage radiation (sometimes % with short term ADT) resulted in 28% of patients biochemically disease free at 5 years; acknowledging that some of these patients had limited follow up after subsequent salvage treatment. In contrast to our initial short-term report, there certainly does appear to be a tail of sustained biochemical failure free (nadir + 0.2) survivors.

A systematic review and meta-analysis of SBRT for metachronous oligometastatic prostate cancer published by Yan et al. [12] reported a median ADT-Free survival of 24.7 months and two-year BF free survival of 33%. ADT-free survival remains a difficult endpoint as the initiation of ADT may be heavily influenced by patient and clinician preference. However, with a median PSA at time of ADT commencement of 5 ug/L, 39% of patients free of any ADT and 55% free of ADT for relapse after 5 years, our cohort appears to have outperformed similar series – many of which used choline PET. This may be due to our approach using PSMA PET guided SBRT, to better exclude poly-metastatic disease[13] and to detect oligometastatic disease at lower serum PSA. It also suggests more rigorous selection of patients for SBRT as well as the repeat application of further SBRT, or more comprehensive nodal RT where appropriate.

The OLI-P study published by Holscher et al. [10] was similar to ours (MDT without ADT for oligometastatic prostate cancer, guided by PSMA PET) and described a very similar failure free survival (22% at 3 years) and a local control rate of 93%. The lower local control rate compared to ours may have been due to lower biologically equivalent dose with 3D conformal RT. With our long follow up (to our knowledge the longest yet published), we were also able to demonstrate that failure free survival rates can be extended with a sustained MDT approach where suitable.

To date, there is no consensus regarding the definition of biochemical response to MDT in oligometastatic disease. We included 2 definitions. The most sensitive is nadir + 0.2 commonly used in post-surgical patients which was our primary endpoint. We also incorporated the Phoenix definition of nadir + 2 which is the ASTRO-RTOG definition following definitive treatment of PCa with RT alone and was also used in SPORT[14] and ORIOLE[7] studies as an a priori secondary endpoint. Given that biochemical failure has little bearing on overall survival [15, 16], this definition may be a more clinically relevant definition of failure following SBRT for oligometastatic disease, as it related more closely to clinical decision making and more closely reflects our ADT avoidance rates (Figs. 2B and 3B).

We also performed an exploratory analysis to see if there was any subgroup that did particularly well, or was unsuitable for, an MDT approach. On univariate analysis no subgroup reached a statistically significant difference.

This is one of few series with moderately large numbers describing long term follow up of SBRT for oligometastatic disease without ADT. The findings are in keeping with well cited literature, including ORIOLE[7] and STOMP[3], further adding to the body of evidence supporting SBRT for oligometastatic disease. At 5 years, our median follow up was similar to STOMP, but far longer than ORIOLE. However, in contra-distinction to these studies, strengths of this study include the routine use of PSMA PET scans for all patients to allow for early detection of oligometastatic disease recurrence as well as assessment of biochemical failure and treatment response. The cohort also consists of patients managed with SBRT alone without concurrent or adjuvant ADT. All patients were treated at a single radiation oncology department, with consistent assessment, dosing, voluming, patient set-up and treatment delivery.

This study has several limitations. It was retrospective and the choice of management of oligometastatic disease was not randomised. Clinician attitudes are likely to have changed over time; in particular, when to offer whole pelvic RT with ADT versus an SBRT approach for “oligo-pelvic” nodal disease. This selection bias is reflected in our median time from definitive treatment to SBRT of 6 years. The lack of a control group (such as patients commenced on ADT at oligometastatic disease detection) also made SBRT evaluation difficult. Furthermore, although median PSA at the time of commencement of ADT for relapse was 5ng/ml, there was no set definition for ADT commencement. Rather, it was a discussion between patient and clinician influenced by PSA doubling time. Finally, although patients treated in the mid to later periods of the study received a lower dose and tighter volume of radiation, this is unlikely to have had a significant impact on outcomes as our local control rates were consistently very high.

Future studies may benefit from uniform dose fractionation schedules and larger patient numbers to allow more rigorous subgroup analysis. Strict patient selection may also improve the effectiveness of this treatment. Longer term analysis and follow up is also required to assess the impact of SBRT MDT and associated ADT avoidance on overall survival.

## Conclusions

PSMA PET guided SBRT for Oligo-metachronous disease is an effective ADT avoidance strategy with a small (15%) but meaningful percentage of patients free of biochemical failure (nadir + 0.2) in the long term. Selected patients with PSMA detected oligometastatic disease should be offered metastasis directed therapy.

## Data Availability

Research data are stored in an Ethics approved (Human Research Ethics Committee) institutional repository and will be shared upon request to the corresponding author.

## Abbreviations

ADT: Androgen deprivation therapy
BF: Biochemical Failure
CTV: Clinical tumour Volume
GTV: Gross tumour volume
MDT: Multi Disciplinary Meeting
PCa: Prostate Cancer
PSA: Prostate Specific Antigen
PSMA PET: Prostate-specific membrane antigen positron emission tomography
RT: Radiation Therapy
SBRT: Stereotactic body radiation therapy
STAD: Short Term Androgen Deprivation
